# Advancing nursing scholarship: the Mozambique model

**DOI:** 10.1080/16549716.2017.1351116

**Published:** 2017-08-03

**Authors:** Judith C. Bruce, Joan Dippenaar, Shelley Schmollgruber, David D. Mphuthi, Agnes Huiskamp

**Affiliations:** ^a^ Department of Nursing Education, Faculty of Health Sciences, University of the Witwatersrand, Johannesburg, South Africa; ^b^ Technical Advisor Training Unit, Health Systems Trust, Durban, South Africa; ^c^ Department of Health Studies, University of South Africa, Pretoria, South Africa

**Keywords:** Capacity development, higher education, nursing, partnership, scholarship

## Abstract

**Background:** Despite the importance of Human Resources for Health for the development and functioning of health systems worldwide, many countries continue to be plagued by poor health systems and a lack of adequate health care. Health systems failures may be attributed to both quantitative and qualitative nursing shortages including the lack of advanced skills to lead health initiatives, to conduct research and to educate other nurses. The response by development partners is usually framed around the production of skilled nurses through the processes of up-skilling and scaling-up. The outcome is expanded practice but with scant attention to the professional advancement of nurses.

**Objectives:** In this paper we present a two-phased capacity development model that adopted professionalization strategies to advance nursing scholarship and consequent postgraduate specialization of the first cohort of nurses in Mozambique. The main objectives were to: develop and implement a clinical course work master’s degree in nursing; and ensure sustainability by capacitating the host institution to continue with the master’s programme following graduation.

**Methods:** Rigorous processes for project discussions, negotiations and monitoring were necessary amid limited resources and a challenging political climate. Forging in-country partnerships, sustaining alliances and government investment are thus key to the success of the Mozambique model.

**Outcomes:** Notwithstanding some difficulties, the process unfolded over a five-year period, graduating the first cohort of 11 senior nurses with a master’s degree, specializing either in critical care and trauma nursing, or maternal and neonatal health.

**Conclusions:** Bridging the skills gap between generalist and specialist nurses is essential for them to manage complex and high acuity cases and to reverse associated morbidity and mortality. We conclude that this model serves as a professionalization strategy to advance nurses’ scholarship of clinical practice, research and teaching.

## Background

The centrality of Human Resources for Health (HRH) to the development and functioning of health systems is acknowledged worldwide; for some countries, it is firmly established in health systems planning. Despite this, many countries continue to be plagued by poor health systems and a lack of health care, particularly in sub-Saharan Africa. As is the case elsewhere in the world, nurses make up the largest category of HRH in sub-Saharan Africa and their role in the promotion of health and the provision of health care at all levels of the health system is undisputed [1]. Health systems failures may be attributed to both quantitative and qualitative nursing shortages, including the lack of advanced skills to lead health initiatives, to conduct research and to educate other nurses. Health initiatives such as the roll-out of mobile health clinics to ensure equitable access to health care are an essential service to rural communities in Mozambique [2]. Mobile health services for the rollout of ARVs are only one example where a critical mass of midwives and neonatal nurses is needed to provide such leadership [3].

Situated in southern Africa and largely a rural country, Mozambique faces some of the most critical human resource problems in the world. The devastating impact of mainly preventable conditions on its people has led to a life expectancy of 51 years and a human development index ranking of 178 out of 187 countries [2]. Among a range of factors, severe HRH constraints are cited as the main contributor to poor health outcomes [4]. The nursing and maternal child health nursing workforce density of 0.32/1000 is below the World Health Organization (WHO) minimum standard of 1/1000 and ranks among the lowest in the world [5]. Mozambique’s burden of disease is due to poverty, infectious diseases (malaria, TB and HIV/AIDS) and, more recently, a surge in injury-related morbidity and mortality. High acuity levels have been recorded with trauma as the second leading cause of death in the 15–59 age group [6]. Compounding factors relate to poor paramedical services and lack of nurses to provide advanced care.

From a quality of education perspective, the Ministry of Health (MOH) is keen to improve the quality and level of nursing staff in line with current evidence that higher qualified nurses are associated with better patient outcomes in hospitals [5]. Nurses, who are the frontline health providers, are often poorly trained and have limited management skills, resulting in weaknesses in policy implementation, coordination and oversight [7]. In selected African countries it was found that nurses lack the capability to perform critical health-care tasks, highlighting the need for relevant curricula and appropriate education of nurses [8].

Several development partners have responded by focusing on the nursing workforce to increase the number of appropriately skilled nurses and midwives. The Nurse Education Partnership Initiative (NEPI) funded by the President’s Emergency Plan for AIDS Relief (PEPFAR) for example, aims to scale up pre-service nursing education programmes to deal with mostly preventable health conditions [8]. Similarly, the International Centre for AIDS Care and Treatment Programs (ICAP) works with the Mozambican MOH to support the production of skilled nurses and midwives and to expand their practice roles. The main aim of the ICAP partnership is to capacitate nurses to deal with the disease burden that their country is facing [7]. Scaling-up and up-skilling of nurses and midwives has become the norm, mainly and correctly so, to deal with countries’ burden of disease. Courses and topics offered are often ‘additive’ and thus supplementary to the mainstream curriculum.

The education of nurses and midwives beyond basic and general nursing programmes in several African countries, including Mozambique, has received little attention. Further education, specialization and professional advancement for Mozambican nurses are non-existent and remain a source of concern [7,9]. Senior nurses, educated via a four-year baccalaureate degree, are assigned by the MOH to rural health services where there is a great need for more skilled and educated nurses [9]. Often nurses take on expanded and complex roles without the necessary training; many experienced nurses are recruited into non-nursing studies, such as the *technicos de medicina* and *technicos de cirurgia*, due to the lack of postgraduate nursing programmes in Mozambique [9]. Professional regulation is lacking due to the absence of a statutory regulatory body for nurses and midwives, leaving the responsibility for nurse education and decisions about professional advancement to the MOH [5]. Lack of further professional training has also been identified as one of the four most frequently cited reasons why African nurses emigrate [10]. Until recently, and with the exception of South Africa, master’s and doctoral programmes have been slow to develop in sub-Saharan Africa [11]. In only a few countries have postgraduate courses been developed leading to specialization in nursing. Although the number of nursing schools in sub-Saharan Africa is fairly substantial, a compounding factor is the lack of nurse educators qualified with a higher degree [11,12]. Prior to this project there were no in-country opportunities for Mozambican nurses to further their education and obtain a nursing specialization, hampering their advancement in the nursing profession.

In this paper we present a different type of capacity development model that adopted professionalization strategies to advance nursing scholarship and consequent postgraduate specialization of the first cohort of nurses in Mozambique. In presenting this work we do not predicate scholarship on a particular conceptual framework or model. Rather we use scholarship to mean those activities and experiences that contribute to the advancement of nursing professionals and the practice of nursing through research and evidence-based practice.

## Context of the project

This capacity development project has its roots in Tanzania, when nurses from universities in East Africa expressed the need for postgraduate education in a clinical nursing specialty within their respective countries [13]. As members of an Africa Honor Society of Nursing, nurse leaders committed to a capacity development project called Collaboration in Higher Education for Nursing and Midwifery in Africa (CHENMA). This resulted in the design of master’s degree programmes for implementation in selected African universities from 2005 onwards. Universities from Southern Africa, namely the universities of Botswana, South Africa, KwaZulu-Natal, Pretoria, Johannesburg and the North West University formed a consortium, partnering with institutions in East Africa. These were: Muhimbili College of Health Sciences and the Kilimanjaro Christian Medical Center in Universities in Tanzania; and Moi, East Africa (Baraton) and Nairobi Universities in Kenya [13]. These institutions became known as the host universities. Building on the successes of CHENMA, the project and funds were extended in 2009 to include Francophone countries: Rwanda and the Democratic Republic of the Congo; and a Lusophone country, Mozambique. Two more South African universities joined the consortium, namely the Universities of the Witwatersrand (Wits) and the Free State.

The objectives of the CHENMA project were to:

- Develop and implement a clinical course work master’s degree in nursing in order to:
- Improve the level of clinical competence in specialized areas of nursing/midwifery
- Equip specialist nurses to conduct clinical and health systems research
- Improve the programme and health service management skills of nurses
- Ensure sustainability by capacitating host universities and training nurse educators.

## The Mozambique model

**Figure 1.**
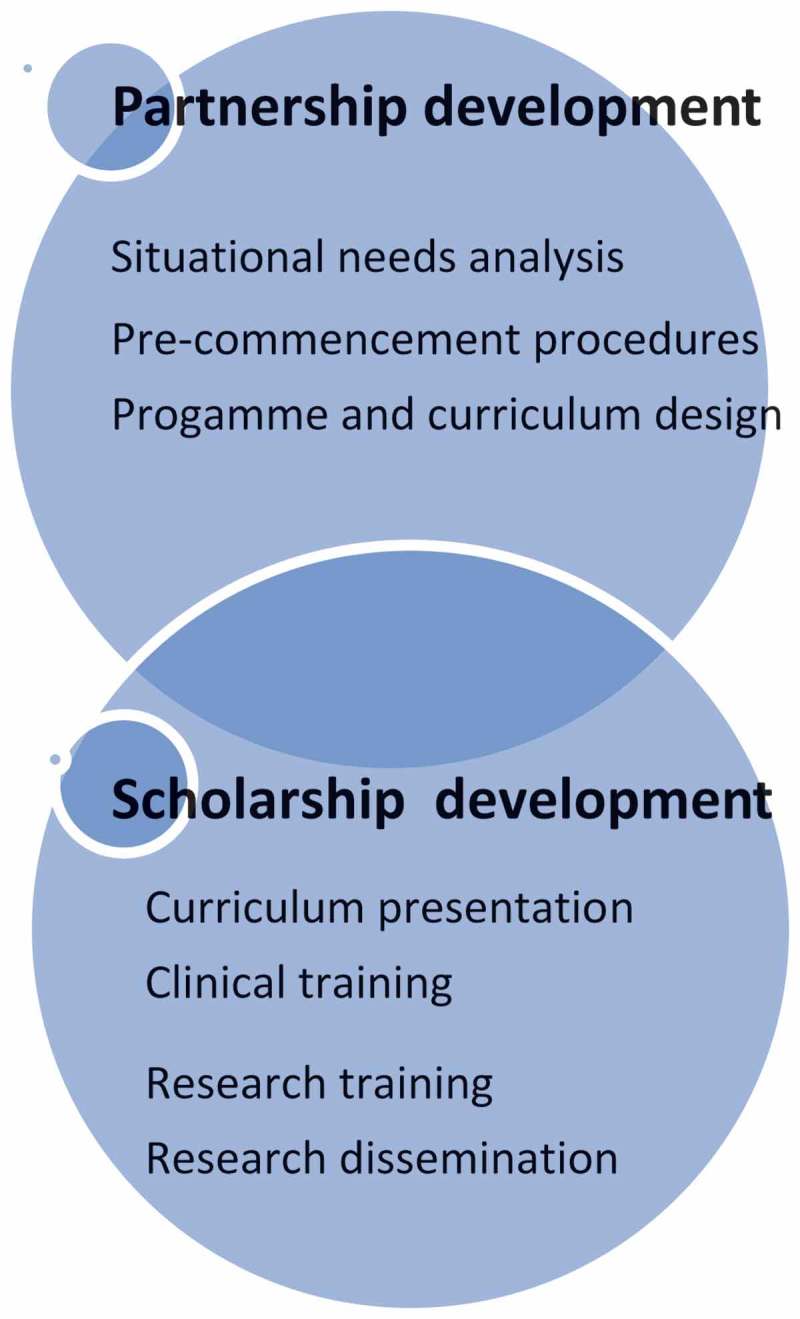
Features of the Mozambique model.

Within the context of the CHENMA project and its objectives, funding was obtained via NEPAD to extend the CHENMA project to Mozambique. Over a five-year period this funding was to be used to develop and implement the Lusophone leg of CHENMA. The Mozambican model for advancing nursing scholarship (Figure 1) was set in two interlinked phases: partnership development and scholarship development with Wits University as the lead consortium university and the Instituto Superior de Ciencias de Saude (ISCISA), the host institution in Maputo, Mozambique.

### Partnership development

**Figure 2.**
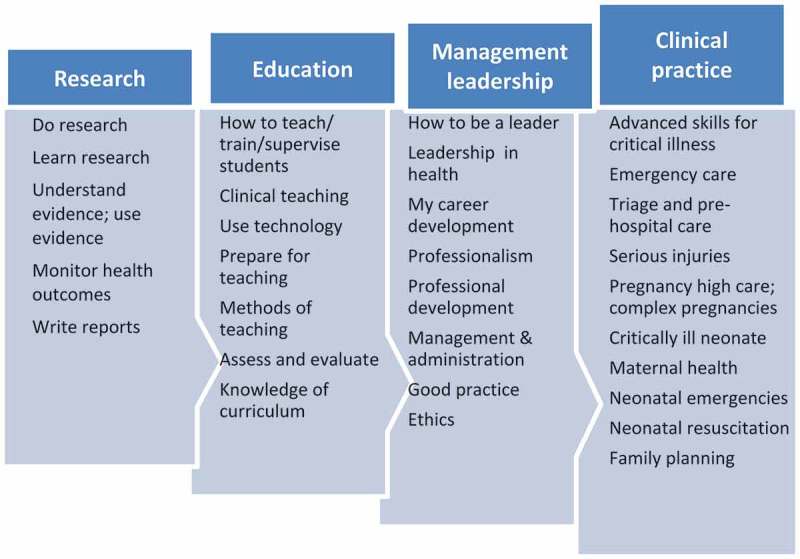
Thematic summary of professional needs analysis.

Partnership and alliance-building is essential for the success of any collaborative venture. The partnership development phase included site visit activities in 2009 with a view to introducing and explaining the purpose of the project during stakeholder meetings, obtaining political buy-in, establishing relationships across countries and between disciplines (nursing and medicine) and developing the programme. The partnering process began with the identification of key stakeholders and holding several meetings with political heads in the Ministries of Health and Education, the Director-General of ISCISA, the Dean of the Faculty of Medicine at Eduardo Mondlane University in Maputo, the management of the Maputo Central Hospital and board members of the Nurses’ Association in Mozambique (ANEMO). Together with a review of relevant literature and key documents a situational analysis was conducted to determine the major health issues as is common practice in similar capacity-building projects in Africa [14,15]. Unsurprisingly, this exercise yielded a similar health profile as outlined in the WHO Country Health System Fact Sheet of 2009. A professional needs analysis was conducted to determine the concomitant education and training needs of nurses. This analysis pointed to a lack of nursing expertise in maternal and neonatal health (MNH) and in critical care and trauma nursing (CCTN). Since these areas have a high degree of conceptual synergy they formed the basis of two electives within the master’s programme. The main needs were arranged in four themes: research; education; management and leadership; and clinical practice (Figure 2).

Government elections at the time and ensuing political uncertainty were some of the difficulties we encountered during partnership and alliance-building. Frequent change in key political heads resulted in an iterative process of partnership development. The programme of activities integral to this phase included the development of a student selection protocol, the development of a clinical master’s programme and the design of a relevant curriculum.

#### Pre-commencement procedures

Prior to commencing the project it was necessary to determine the selection criteria for the master’s programme as a joint venture between the consortium and host institutions; the actual selection of candidates was done collaboratively by ISCISA and the MOH through the respective hospitals. Candidates were purposefully selected to cover three provinces: Zambezia, Sofala and Maputo, which have the country’s largest hospitals. To be selected, candidates must have completed a four-year bachelor’s degree in nursing and be practising as a nurse or midwife in an area closely aligned to a clinical elective. A total of 12 students were selected: seven in MNH and five in CCTN.

It was also necessary to strengthen the host institution’s training capacity and resources to offer the master’s programme. This involved mainly the appointment and orientation of teaching translators, strengthening information technology (IT) and connectivity, and the purchasing of audio-visual equipment, including laptop computers and a data projector. Since nursing specialization was lacking there were no nurses qualified to act as clinical mentors or supervisors for the specialist clinical training component of the master’s degree. In the absence of senior nurses trained in specialized skills, we drew on the clinical expertise of medical practitioners in partnering hospitals who committed to assist with clinical training and supervision. Additionally, three senior nurses from Maputo were trained as clinical supervisors at the Charlotte Maxeke Johannesburg Academic Hospital over a four-week block.

#### Programme and curriculum design

The master’s programme was designed to include course work and a research report over a two-year part-time basis. A block system was used to present the core programme strands, namely, theory, clinical and research. As agreed to by the MOH, nurses were released from the workplace during blocks of two or four weeks (40 hours per week) with clinical placements in the workplace in between blocks. A total of 14 teaching and research blocks were offered. A competency-based approach informed curriculum design with core and elective modules as the units of learning, guided by competencies to be achieved at modular and programme exit levels.

### Scholarship development

Scholarship development is what distinguishes this capacity development project from others. The purpose of this phase was to enable students to embark on a journey of nursing scholarship that is as complete as possible and equivalent to global standards. This phase of developing nursing capacity aimed at instilling in students a culture of inquiry from the outset of the course during theory contact sessions. It began with student engagement in research, culminating in publicizing their research.

**Figure 3.**
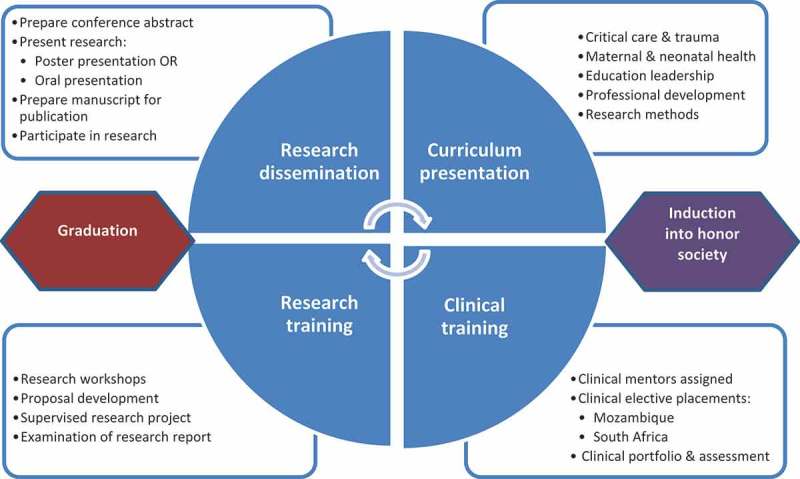
The scholarship journey.

Scholarship development was comprised of four signposts, namely: curriculum presentation; clinical training; research training; and research dissemination, depicted as the scholarship journey in Figure 3. These signposts served as non-linear indicators of students’ learning requirements and not their progression in the programme.

#### Curriculum presentation

The theoretical component of the curriculum was presented at ISCISA by nurse academics from the consortium universities. Pedagogical approaches included core lectures, group discussions, case studies, paper-based problem-solving exercises (PSEs), individual assignments and tutorials. Although we aspired to using online and blended learning we encountered constraints in the IT capabilities of the host institution; however, we capitalized on initiatives such as Hinari to afford students electronic access to scientific literature. Formative and summative assessments were aligned with the module outcomes and integrated into each teaching approach. As required by both the host and consortium institutions, the language of instruction was English; a translator was used during all classroom interactions since all students were Portuguese-speaking. A post-course review indicates that the students preferred to be taught in English as this opens opportunities to publish and to enrol in a doctoral programme [16].

The curriculum was comprised of two elective courses and three compulsory courses, each elective having three modules. For the *maternal and neonatal health* elective the modules included midwifery care and systems, reproductive health and neonatal high care. Modules in the *critical care and trauma nursing* elective were: Essentials of Critical Care Nursing, Applied Critical Care Nursing, and Accidents and Emergencies. Within the education leadership course there were two modules, which aimed at developing postgraduate nurses’ education capacity to teach future senior nurses and to prepare them for academic positions at the host institution. Some topics included curriculum development, educational theory and pedagogy, teaching, learning and assessment. The module in the professional development course aimed at preparing nurses for the roles of a specialist practitioner, namely, leader, educator, clinician, manager and consultant. The research course provided training in research methods and doing a research project. We discuss these under research training.

Once the first-year curriculum had been successfully completed, students were nominated to be inducted under the category ‘nurse leader’ into a global honor society of nursing, Sigma Theta Tau International (STTI). The purpose was for them as scholars and emerging researchers to develop a global mindset and engage with a global community of nurses and midwives to advance the health of communities and society at large. Membership of STTI recognizes nursing excellence and provides further opportunity for nurses to grow as researchers, obtain grants and scholarships, advance their leadership skills and expand their sphere of influence.

#### Clinical training

Although diagrammatically illustrated in a stepwise manner, clinical training was interspersed between theory blocks and formed an integral part of the students’ work day. The placement of students in clinical areas aligned with their elective was thus essential for the achievement of clinical competencies. Clinical training and supervision was done by doctors and senior nurses in the areas of specialization, and less by lecturers during block times. The biggest challenge in clinical training was twofold: integration with theory was almost impossible due to the unavailability of local advanced learning opportunities. Where these were available, students often felt that not enough time was allocated to learn new skills [16]. The second challenge was the mismatch between what was taught as the ideal and what was available to them in practice. This applied particularly to advanced equipment and treatment modalities lacking in major health settings in Mozambique.

Clinical training took place in clinical (simulation) laboratories, high care and intensive care units of the Maputo Central Hospital (Maputo province), Quelimane Hospital (Zambezia province) and Beira Hospital (Sofala province). A noteworthy feature is an intensive period of clinical internship at the Charlotte Maxeke Johannesburg Academic Hospital in South Africa, which was an experiential learning highlight for the students. Paradoxically, it was also a source of great dissatisfaction among students due to its short duration and language barriers encountered [16]. Formative and summative clinical assessments were conducted to progress students and to determine clinical competence.

#### Research training

Developing students’ research skills involved research workshops and a series of lectures, group discussions and PSEs to enable mastery of research methods. We ran a total of five research workshops, varying between three to five days, to augment the research theory block. The purpose of these workshops was to progress students towards achieving their research milestones: conceptualizing a research project; developing a research proposal; obtaining ethical clearance; reporting on project implementation; and presenting preliminary findings. Ancillary skills acquired during these milestones include, among others, putting forward and defending a viewpoint, giving and receiving critique, communicating verbally and preparing a PowerPoint presentation.

Research projects were conducted in their field of specialization under the supervision of a local research supervisor sourced mainly from medical practitioners with an appropriate postgraduate qualification. On completion of their projects students were required to submit a research report for examination by two examiners (internal and external), selected and appointed by the consortium university; external examiners were sourced from South African universities.

Eleven students (n = 11; 91.2%) graduated in 2015 upon successful completion of both course work and research components; one student is in the process of completing the research component.

#### Research dissemination

In this model we conceptualized scholarship of research as work that is made public and open to peer scrutiny, and for postgraduate students the research dissemination stage was the conduit to do so. Scholarship also means scholarly work that is reproducible and can be built on by other scholars [17]. Used since the late nineteenth century, peer review has become common practice in the scientific community and is an essential part of the scholarship journey. Due to the growing volume of research being produced and disseminated, the need for external peer scrutiny has become essential [18]. Because peer review processes have become so closely aligned with notions of quality [19], we also thought of it as a proxy for the quality of students’ research.

Graduates from this project were required to prepare a conference abstract for either an oral or poster presentation at an international scientific conference. To date four graduates (n = 4; 36.4%) have presented their research at international conferences and in the case of all graduates, manuscript writing is in process. Although none of the graduates have become involved in other research projects, the majority have begun to frame practice-based questions that could lead to further research. We anticipate that graduates’ self-initiated research will grow and may precipitate their participation in collaborative research projects.

## Discussion

This model has capacitated the host institution, ISCISA, by providing baseline resources and academic support to implement the first ever master’s degree programme for nurses and midwives in Mozambique. The difference the Mozambique model makes can be described on several levels. Firstly, it is significant for the history of nursing in Mozambique and contributes to an upward turn in the development of nursing specialization, which was previously non-existent. Bridging the skills gap between generalist and specialist nurses is essential for them to manage complex and high acuity cases and to reverse associated morbidity and mortality. Secondly, postgraduate study opportunities also pave the way for developing nurse leaders who are empowered to use knowledge and evidence in their practice and to engage in scholarship in its broadest sense. Thirdly, having had cross-country learning opportunities nurses and midwives in the master’s programme were exposed to international trends and best practices, expressing the desire to lobby for change and improvement in their own health-care settings.

By adopting elements of ‘train-the-trainer’ approach the Mozambique model has provided a cadre of nurse educators who will continue with the training of nurses for critical care, trauma, and maternal and neonatal health services. Building academic leadership and education capacity among nurses creates a pool of nurses and midwives to design nursing curricula, conduct their own examinations and make judgements about competency standards for the profession.

Addressing the challenges associated with capacity-building is essential to provide direction for the future and to ensure sustainability of this model. There are several challenges to navigate in order to sustain the advancement of nurses and nursing in Mozambique and to gain momentum in their attempts towards professional independence. Medical dominance persists and is considered to be one of the main obstacles in the nurses’ quest for professional autonomy and self-regulation. The lack of human resources for health policies and planning systems also contribute to instances where nurses’ attempts towards advancement and professionalization are at odds with the strategic interests of the health sector. This in turn gives rise to two colliding narratives about the nursing profession. Leveraging the efforts of development partners and collaborators will contribute towards establishing a regulatory authority for nurses and midwives in Mozambique. The final hurdle is in the parliament and clearing it lies in the hands of nurses.

With regard to education, we acknowledge that the medical model is the preferred model for the training of nurses and midwives in this region, as it is for large parts of sub-Saharan Africa. However, for meaningful nursing practice nurses and midwives need exposure to nursing theories and their application to the art and science of nursing. New and transformative ways of learning are rapidly evolving and are replacing traditional models of education, which will more readily be taken up by a new generation of nurse educators. By producing better qualified practitioners, such as the graduates from this project, we anticipate that they will use progressive and transformative approaches to educate the future generation of nurses. This anticipated outcome and those associated with clinical specialization will drive a review of the project within the next five years.

From the outset the underpinning values of the partnership – trust, quality and equality – were considered key for the implementation and completion of the Mozambique leg of CHENMA. Its success depended to a large extent on these values and on the principles of philanthropy, volunteerism, commitment and reciprocity. The host institution’s ability to sustain the output of specialist nurses will depend on whether the Mozambican government’s investment in nursing will continue into the future.

## Conclusion

This model has had a profound impact on the professional development of nurses in Mozambique. It provides a template to address the professional advancement gap and to strengthen specialist nursing and midwifery skills needed in a variety of practice settings. The interlinked phases of our model serve as a professionalization strategy to advance nurses’ scholarship of clinical practice, research and teaching. Forging in-country partnerships and sustaining alliances in practice environments, though, are key to the success of the Mozambique model.

## Paper context

In this manuscript we provide methodological and contextual insights into a five-year postgraduate nursing capacity development model in Mozambique. It is well known that contemporary nursing capacity development focuses on pre-service and in-service education to expand nurses’ skills to address major health challenges. The Mozambique model combines skills development with professionalization strategies to advance nurses’ scholarship of clinical practice, research and teaching. The ability of the host institution to sustain the output of specialist nurses depends on the government’s investment in the future of nursing.
